# British Columbia’s Safer Opioid Supply Policy and Opioid Outcomes

**DOI:** 10.1001/jamainternmed.2023.7570

**Published:** 2024-01-16

**Authors:** Hai V. Nguyen, Shweta Mital, Shawn Bugden, Emma E. McGinty

**Affiliations:** 1 School of Pharmacy, Memorial University, St John’s, Newfoundland and Labrador, Canada; 2 College of Pharmacy, University of Manitoba, Winnipeg, Manitoba, Canada; 3 Weill Cornell Medicine, New York, New York

## Abstract

**Importance:**

In March 2020, British Columbia, Canada, became the first jurisdiction globally to launch a large-scale provincewide safer supply policy. The policy allowed individuals with opioid use disorder at high risk of overdose or poisoning to receive pharmaceutical-grade opioids prescribed by a physician or nurse practitioner, but to date, opioid-related outcomes after policy implementation have not been explored.

**Objective:**

To investigate the association of British Columbia’s Safer Opioid Supply policy with opioid prescribing and opioid-related health outcomes.

**Design, Setting, and Participants:**

This cohort study used quarterly province-level data from quarter 1 of 2016 (January 1, 2016) to quarter 1 of 2022 (March 31, 2022), from British Columbia, where the Safer Opioid Supply policy was implemented, and Manitoba and Saskatchewan, where the policy was not implemented (comparison provinces).

**Exposure:**

Safer Opioid Supply policy implemented in British Columbia in March 2020.

**Main Outcomes and Measures:**

The main outcomes were rates of prescriptions, claimants, and prescribers of opioids targeted by the Safer Opioid Supply policy (hydromorphone, morphine, oxycodone, and fentanyl); opioid-related poisoning hospitalizations; and deaths from apparent opioid toxicity. Difference-in-differences analysis was used to compare changes in outcomes before and after policy implementation in British Columbia with those in the comparison provinces.

**Results:**

The Safer Opioid Supply policy was associated with statistically significant increases in rates of opioid prescriptions (2619.6 per 100 000 population; 95% CI, 1322.1-3917.0 per 100 000 population; *P* < .001) and claimants (176.4 per 100 000 population; 95% CI, 33.5-319.4 per 100 000 population; *P* = .02). There was no significant change in prescribers (15.7 per 100 000 population; 95% CI, −0.2 to 31.6 per 100 000 population; *P* = .053). However, the opioid-related poisoning hospitalization rate increased by 3.2 per 100 000 population (95% CI, 0.9-5.6 per 100 000 population; *P* = .01) after policy implementation. There were no statistically significant changes in deaths from apparent opioid toxicity (1.6 per 100 000 population; 95% CI, −1.3 to 4.5 per 100 000 population; *P* = .26).

**Conclusions and Relevance:**

Two years after its launch, the Safer Opioid Supply policy in British Columbia was associated with higher rates of safer supply opioid prescribing but also with a significant increase in opioid-related poisoning hospitalizations. These findings will help inform ongoing debates about this policy not only in British Columbia but also in other jurisdictions that are contemplating it.

## Key Points

**Question:**

Was there an association of British Columbia’s Safer Opioid Supply policy with opioid prescribing and opioid-related health outcomes in the first 2 years of implementation?

**Findings:**

In this cohort study using the difference-in-differences method, the Safer Opioid Supply policy was associated with a large increase in the number of opioid prescriptions dispensed, a moderate increase in the number of individuals with at least 1 opioid prescription dispensed, and a substantial increase in opioid-related poisoning hospitalizations.

**Meaning:**

Two years after its launch, the Safer Opioid Supply policy was associated with greater prescribing of safer supply opioids but also with a significant increase in opioid-related poisoning hospitalizations.

## Teaser

This cohort study examines whether there were changes in opioid prescribing and opioid-related health outcomes after implementation of British Columbia’s Safer Opioid Supply policy.

## Introduction

Canada’s opioid crisis has accelerated markedly in recent years. In 2022, 14 opioid-related poisoning hospitalizations occurred each day, while 20 people per day died of an overdose on average.^1^ With potent synthetic opioids from the unregulated market fueling this crisis, there is growing interest in offering a safe supply of regulated, pharmaceutical-grade opioids to people who use drugs to help reduce the risk of overdose and poisonings.^2^

In March 2020, British Columbia became the first jurisdiction globally to launch a provincewide Safer Opioid Supply policy that allows individuals at high risk of overdose to receive pharmaceutical-grade opioids free of charge prescribed by a physician or nurse practitioner.^3^ This policy initially covered select opioids (hydromorphone and sustained-release oral morphine),^3^ and in July 2021, it was made permanent and expanded to include additional drugs, including injectable fentanyl.^3,4^ In June 2023, 4619 people were prescribed safer supply opioid medications.^5^ Outside British Columbia, there have also been a limited number of federally funded pilot safer supply programs in cities in Ontario, Quebec, and New Brunswick since 2020.^6,7^

While this harm-reduction policy is intended to reduce overdose or poisoning risks and remove barriers to care access for people who use drugs,^8^ some people suggest that the limited range of lower-potency opioids available through safer supply programs may not meet the needs of people who use drugs and are accustomed to high-potency opioids.^9^ Furthermore, there are concerns that providing a safer supply could discourage people who use drugs from receiving proven substance use treatment and encourage potential diversion of prescribed opioids.^8,10^ In a recent debate in the House of Commons, opposition parties blamed the Safer Opioid Supply policy for continued opioid overdose deaths in British Columbia, arguing that opioids obtained from the program are being traded for fentanyl-laced opioids on the unregulated market.^11,12^

There is little evidence on the impacts of safer supply programs to inform this debate. Some qualitative studies suggest that safer supply programs positively impacted the lives of participants^13^ and were associated with reduced overdose risks,^14,15^ although participants highlighted inadequate substitutability of hydromorphone for unregulated opioids.^16^ Three quantitative studies found that the programs were associated with lower risk of nonfatal overdose.^17,18,19^ However, these studies looked at only small-scale programs adopted in specific community settings (COVID-19 isolation shelters and primary health care centers) in Nova Scotia and Ontario, lacked control groups^17,18^ or had a control group that was not comparable with the treated group,^19^ and did not satisfactorily account for other concurrent interventions and policies.^17,18,19^ Using a quasi-experimental difference-in-differences (DD) design and administrative data from 3 Canadian provinces, this study sought to overcome the limitations of prior studies and provide the first evidence, to our knowledge, on the association of British Columbia’s Safer Opioid Supply policy with opioid prescribing and opioid-related health outcomes.

## Methods

### Study Design

In this cohort study, we used the DD method^20,21,22,23,24^ to compare prepolicy to postpolicy changes in outcomes in British Columbia (where the Safer Opioid Supply policy was implemented) with similar changes in comparison provinces (Manitoba and Saskatchewan) that did not implement this policy. The key assumption underlying the DD analyses was that, in the absence of the policy, the trends in outcomes in British Columbia would be similar to the trends in the comparison provinces. Our study used deidentified, aggregate province-level data; hence, no ethics approval or informed consent was required, as per Newfoundland and Labrador’s Health Research Ethics Board guidelines. This study followed the Strengthening the Reporting of Observational Studies in Epidemiology (STROBE) reporting guideline.

### Outcomes, Data, and Study Period

Opioid prescribing outcomes included number of opioid prescriptions dispensed, number of people with at least 1 opioid prescription dispensed (hereafter, *opioid claimants*), and number of opioid prescribers, measured per 100 000 population. All outcomes were specific to the types of opioids targeted by British Columbia’s Safer Supply policy (ie, hydromorphone, morphine, fentanyl, and oxycodone). While these types of opioids were targeted by the policy, these were not exclusively safer supply products and had been prescribed for pain management and other clinical reasons before the policy was implemented. Opioid-related health outcomes included rates of opioid overdose poisoning hospitalizations (hereafter, *hospitalizations*) and deaths from apparent opioid toxicity (hereafter, *deaths*). All data were at the province quarter level.

Data on opioid prescriptions were obtained from the Canadian National Prescription Drug Utilization Information System Database. Prescription data included all claims (both public and private) dispensed in community pharmacies and were categorized at Anatomical Therapeutic Chemical level 5 (all forms of fentanyl, hydromorphone, oxycodone, and morphine). Hospitalizations were identified using *International Statistical Classification of Diseases and Related Health Problems, Tenth Revision* codes T40.0, T40.1, T40.2, T40.3, T40.4, and T40.6 and included both unintentional and intentional poisonings.^25,26^ Deaths were defined as “a death caused by intoxication/toxicity (poisoning) resulting from substance use, where 1 or more of the substances is an opioid, regardless of how it was obtained (eg, illegally or through personal prescription).”^25^ Data on hospitalizations and deaths were publicly available from the Public Health Agency of Canada.^25^ Given the data availability, our study period spanned from quarter 1 of 2016 (January 1, 2016) to quarter 1 of 2022 (March 31, 2022).

### Comparison Provinces

Our study used Manitoba and Saskatchewan as provinces for comparison with British Columbia. These 2 provinces did not implement the policy and, in addition to British Columbia, were the only provinces that had data for both opioid prescriptions (across all ages) and opioid-related health outcomes.

### Statistical Analysis

We estimated DD regressions using province quarter-level data. The covariate of interest was an indicator for the Safer Opioid Supply policy, which was equal to 1 if the policy was in effect in a province (ie, in British Columbia after quarter 1 of 2020) and 0 otherwise. These analyses controlled for time-varying province-level covariates, including proportion of individuals aged 0 to 17 years, proportion of males, Consumer Price Index, and unemployment rate as well as public health COVID-19 restrictions (using the COVID-19 stringency index developed by Bank of Canada^27^). The regressions also included province indicators to control for all time-invariant characteristics of provinces and quarter-year indicators to control for secular changes or shocks in outcomes that are common to British Columbia and the comparison provinces. Additionally, we included province-specific linear time trends to control for possible differences in trends across provinces.

We estimated the regressions by ordinary least squares and calculated heteroskedasticity-consistent HC3 SEs. All analyses were conducted using Stata, version 18 (StataCorp LLC). Tests were 2 sided, and a significance level of *P* < .05 was used.

We conducted several analyses to investigate the robustness of our results. As the policy’s launch coincided with the onset of the COVID-19 pandemic, we reran the analysis excluding the COVID-19 washout period between quarter 2 of 2020 and quarter 1 of 2021 and then examined the policy effects separately during the first year (ie, the policy’s launch) and the second year (ie, the policy’s expansion) for a dose-response relationship.

We also examined the sensitivity of our results to exclusion of province-specific linear time trend, demographic controls, and the COVID-19 stringency index and examined sensitivity of results to choice of comparison provinces by additionally including Alberta and Nova Scotia as comparison provinces. Next, we conducted an event study analysis to assess the validity of the parallel trends assumption. Finally, to examine the robustness of our results to potential violation of the parallel trends assumption, we used the synthetic DD method^28^ that reweights control groups and time periods to ensure that outcome trends are parallel between control and treatment groups. This analysis included not only Manitoba, Saskatchewan, Alberta, and Nova Scotia but also Ontario and New Brunswick as comparison provinces. Further details of the statistical analysis are provided in the eMethods in Supplement 1.

We also examined how changes in prescription outcomes associated with the Safer Opioid Supply policy varied by age and sex of claimants. Subgroup analyses for health outcomes were not feasible as subgroup data on these outcomes were not available at the province quarter level.

## Results

### Descriptive Statistics

**Figure 1.**
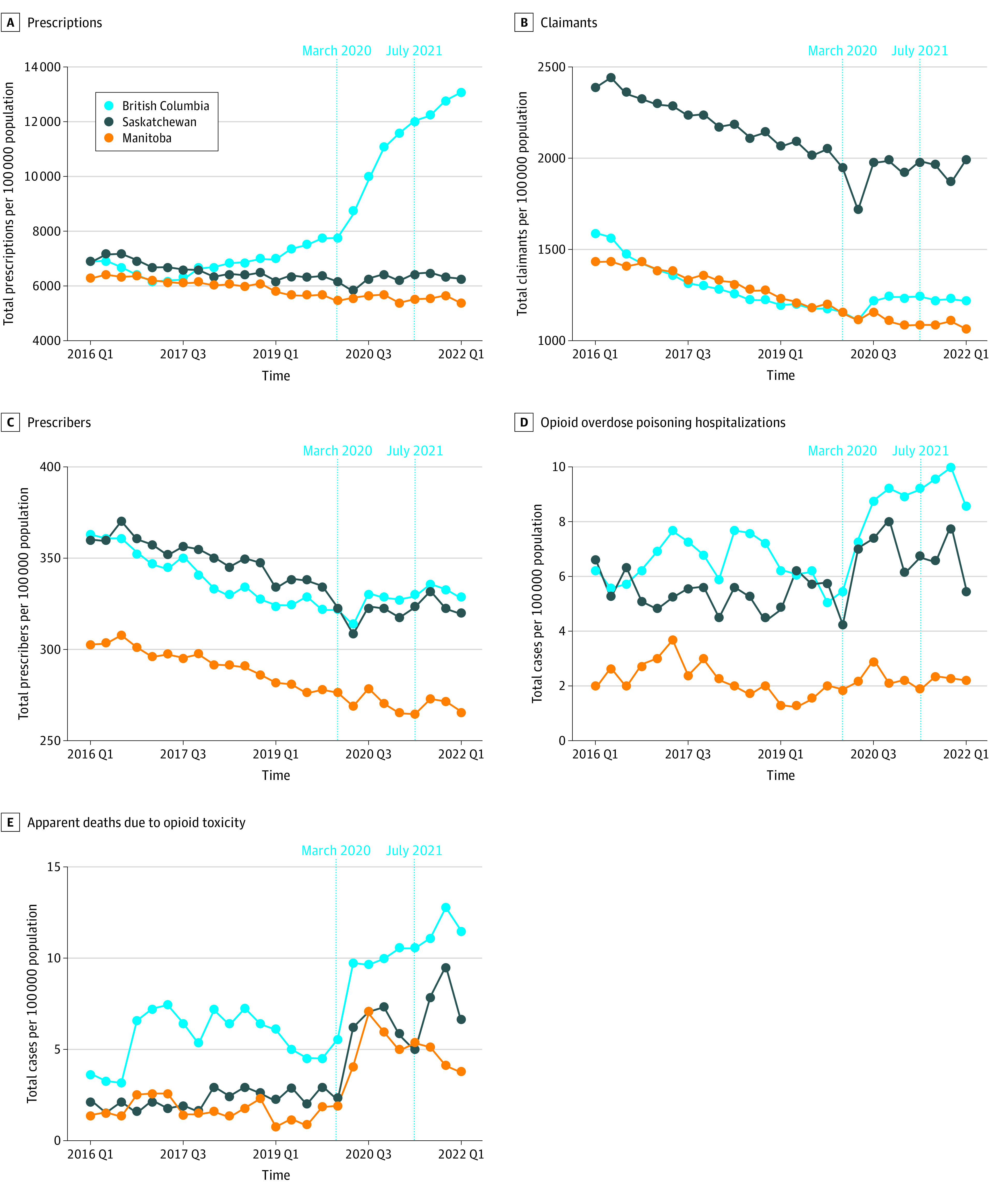
Unadjusted Trends in Opioid-Related Outcomes in British Columbia, Canada, and Comparison Provinces From Quarter (Q) 1 of 2016 to Q1 of 2022 Prescription data are based on claims for all opioid types for all age groups obtained from the National Prescription Drug Utilization Information System. Data on opioid-related hospitalizations and deaths from apparent opioid toxicity are from the Public Health Agency of Canada 2016 to 2022. Control provinces were Manitoba and Saskatchewan. The Safer Opioid Supply policy was implemented in March 2020 and expanded in July 2021.

Figure 1 shows the outcome trends. In Manitoba and Saskatchewan, opioid prescriptions remained stable throughout the study period. In British Columbia, there was a gradual, small increase in opioid prescriptions (from 6227.1 per 100 000 population in quarter 3 of 2017 to 7750.4 per 100 000 population in quarter 1 of 2020) before implementation of the Safer Opioid Supply policy. This increase, however, accelerated sharply after the policy’s implementation. The number of prescriptions increased by 52% from 8598.6 per 100 000 population in quarter 2 of 2020 to 13 070.1 per 100 000 population in quarter 1 of 2022. The trends for the opioid claimant outcome declined for the comparison provinces throughout the study period. Meanwhile, in British Columbia, these trends declined before the policy but began to increase after policy implementation. For the number of prescribers, there was a declining trend in all 3 provinces before the policy. After the policy was implemented, the number of prescribers continued to decline in Manitoba, while there was an upward trend in Saskatchewan and British Columbia.

In all 3 provinces, hospitalizations fluctuated from 1 quarter to the next before the policy was implemented. However, the trend appeared to be similar between British Columbia and the comparison provinces. After policy implementation in March 2020, there was a sharp increase in hospitalizations in British Columbia that continued until quarter 4 of 2021. An increase in hospitalizations was observed in Saskatchewan after the policy, but this increase was followed by declines and periods of no change—a pattern more consistent with quarter-by-quarter fluctuations than an upward trend. The postpolicy trend for hospitalizations in Manitoba was similar to the prepolicy trend and was relatively flat. A spike in death rates was observed in British Columbia at the time of policy implementation. Similar spikes were seen in Saskatchewan and Manitoba, but unlike in British Columbia, these increases reversed in the subsequent quarters.

### Regression Results

Table 1 presents the results from the main analyses. The Safer Opioid Supply policy was associated with an increase of 2619.6 prescriptions per 100 000 population (95% CI, 1322.1-3917.0 per 100 000 population; *P* < .001) in British Columbia compared with the corresponding increase in the comparison provinces. This increase represented a 34% relative increase compared with 7730 prescriptions per 100 000 population in British Columbia in quarter 4 of 2019 (before the policy was implemented). The policy was also associated with statistically significant increases in the number of claimants of opioids (176.4 per 100 000 population; 95% CI, 33.5-319.4 per 100 000 population; *P* = .02). There was no significant change in the number of prescribers of opioids (15.7 per 100 000 population; 95% CI, −0.2 to 31.6 per 100 000 population; *P* = .053) covered under the policy. Subgroup analyses by age and sex revealed that the large increases in opioid prescriptions and opioid claimants were driven by males and those aged 25 to 64 years (eTable in Supplement 1).

**Table 1.** Outcome Changes Associated With the Safer Opioid Supply Policy^a^

Outcome (province-quarter data points)	Difference-in-differences estimates, per 100 000 population (95% CI)	*P* value
Prescription rate (n = 75)	2619.6 (1322.1 to 3917.0)	<.001
Claimant rate (n = 75)	176.4 (33.5 to 319.4)	.02
Prescriber rate (n = 75)	15.7 (−0.2 to 31.6)	.053
Hospitalization rate (n = 75)	3.2 (0.9 to 5.6)	.01
Death rate (n = 75)	1.6 (−1.3 to 4.5)	.26

^a^ Data are from quarter 1 of 2016 to quarter 1 of 2022. Estimates are from difference-in-differences regressions estimated using ordinary least squares and controlled for proportion of individuals aged 0 to 17 years in the population, proportion of males, Consumer Price Index, unemployment rate, and COVID-19 restriction score in the province, province and quarter-year fixed effects, and province-specific linear time trend. Comparison provinces were Manitoba and Saskatchewan. Heteroskedasticity-consistent HC3 SEs were used.

The hospitalization rate increased by 3.2 per 100 000 population (95% CI, 0.9-5.6 per 100 000 population; *P* = .01) after policy implementation in British Columbia compared with the corresponding increase in the comparison provinces. This represented a 63% relative increase compared with 5.7 hospitalizations per 100 000 population in quarter 4 of 2019. Meanwhile, there was no statistically significant change in deaths (1.6 per 100 000 population; 95% CI, −1.3 to 4.5 per 100 000 population; *P* = .26; a 36% relative increase compared with 4.5 per 100 000 population in quarter 4 of 2019).

**Figure 2.**
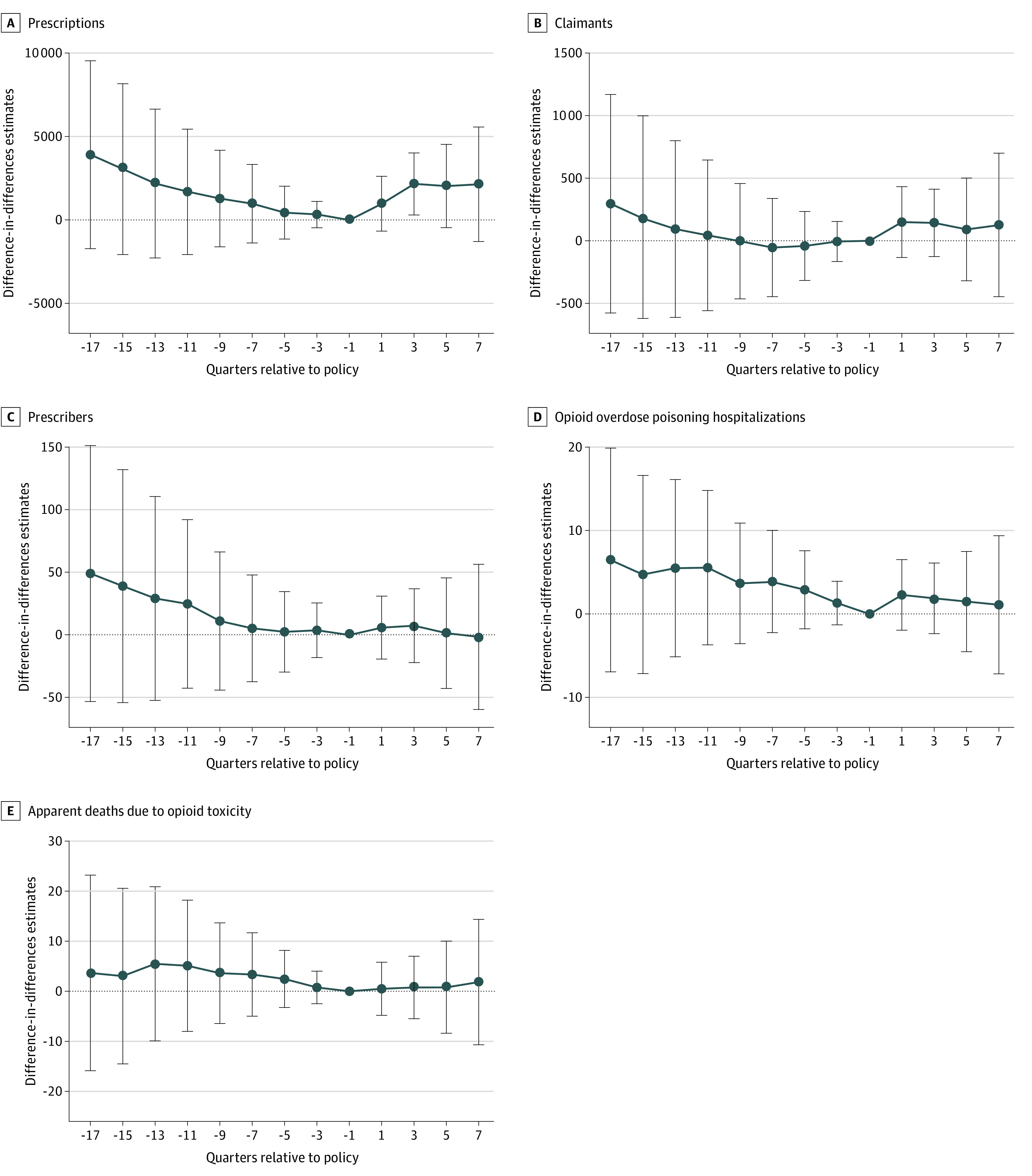
Quarterly Differences in Outcomes Between British Columbia, Canada, and the Comparison Provinces Before and After Safer Opioid Supply Policy Implementation Difference-in-differences estimates (95% CIs) are shown. The comparison provinces were Manitoba and Saskatchewan. Time 0 represents the quarter after the Safer Opioid Supply policy was first implemented (quarter 2 of 2020). All regressions controlled for province-specific linear time trend. Error bars indicate 95% CIs.

Table 2 reports the sensitivity analyses. When we included separate indicators for policy introduction and expansion, the increases in both prescriptions and hospitalizations were larger during the policy expansion phase than during the introduction phase. The DD estimates were also larger when we excluded the COVID-19 washout period. Our results were also robust to the exclusion of control variables for demographic factors, province-specific linear time trend, and the COVID-19 stringency index. When we expanded the control group to include 4 provinces (Manitoba, Saskatchewan, Alberta, and Nova Scotia) and then 6 provinces (Manitoba, Saskatchewan, Alberta, Nova Scotia, Ontario, and New Brunswick), we also obtained evidence of an increase in hospitalizations, both graphically (eFigures 1 and 2 in Supplement 1) and in the regression analyses. In these analyses, the increases in deaths were smaller and remained statistically insignificant. Finally, despite the slight difference in prepolicy trends in the number of prescriptions observed in Figure 1, the event study indicated no systematic differences in prepolicy trends between British Columbia and the comparison provinces for any outcome (Figure 2), suggesting that inclusion of province-specific linear time trends helped satisfy the parallel trends assumption.

**Table 2.** Sensitivity Analyses^a^

Outcome	Difference-in-differences estimates, per 100 000 population (95% CI)	*P* value
**Addressing confounding effects of COVID-19 pandemic**		
Policy introduction and expansion (n = 75)		
Prescription rate		
Introduction	2624.6 (1193.6 to 4055.6)	.001
Expansion	3418.5 (1848.1 to 4988.9)	.001
Claimant rate		
Introduction	176.7 (27.6 to 325.7)	.02
Expansion	211.4 (21.1 to 401.8)	.03
Prescriber rate		
Introduction	15.7 (−0.9 to 32.3)	.06
Expansion	19.8 (1.8 to 37.9)	.03
Hospitalization rate		
Introduction	3.2 (1.0 to 5.4)	.006
Expansion	4.3 (1.3 to 7.3)	.006
Death rate		
Introduction	1.6 (−1.2 to 4.4)	.24
Expansion	3.0 (−1.6 to 7.5)	.19
Excluding quarter 2 of 2020 to quarter 1 of 2021 (n = 63)		
Prescription rate	4215.1 (3656.3 to 4773.9)	<.001
Claimant rate	157.7 (53.9 to 261.4)	.004
Prescriber rate	20.7 (10.1 to 31.4)	<.001
Hospitalization rate	3.6 (1.7 to 5.5)	<.001
Death rate	2.5 (−0.7 to 5.8)	.12
**Alternative regression specifications**		
Excluding linear time trend (n = 75)		
Prescription rate	4211.7 (3111.1 to 5312.3)	<.001
Claimant rate	156.8 (82.1 to 231.5)	<.001
Prescriber rate	15.3 (6.4 to 24.2)	.001
Hospitalization rate	3.2 (1.9 to 4.5)	<.001
Death rate	1.4 (−0.5 to 3.3)	.15
Excluding demographic covariates (n = 75)		
Prescription rate	3022.7 (1999.5 to 4045.8)	<.001
Claimant rate	186.2 (57.1 to 315.3)	.006
Prescriber rate	19.2 (6.4 to 32.1)	.004
Hospitalization rate	2.9 (1.0 to 4.8)	.003
Death rate	1.5 (−1.1 to 4.1)	.25
Excluding COVID-19 stringency index (n = 75)		
Prescription rate	2621.2 (1324.1 to 3918.3)	<.001
Claimant rate	175.7 (29.7 to 321.7)	.02
Prescriber rate	15.7 (−0.008 to 31.3)	.050
Hospitalization rate	3.2 (1.0 to 5.4)	.006
Death rate	1.6 (−1.1 to 4.3)	.24
**Expanded sets of comparison provinces**		
Difference-in-differences with 4 comparison provinces (n = 125)^b^		
Hospitalization rate	2.4 (0.5 to 4.3)	.01
Death rate	1.2 (−0.7 to 3.2)	.21
Synthetic difference-in-differences with 6 comparison provinces^c^		
Hospitalization rate	2.0 (0.6 to 3.4)	.007
Death rate	0.4 (−4.8 to 5.5)	.89

^a^ Data are from quarter 1 of 2016 to quarter 1 of 2022 (unless stated otherwise); n values represent the number of observations. Estimates are from difference-in-differences regressions estimated using ordinary least squares and controlled for proportion of individuals aged 0 to 17 years in the population, proportion of males, Consumer Price Index, unemployment rate, and COVID-19 restriction score in the province, province and quarter-year fixed effects, and province-specific linear time trend (unless stated otherwise). Comparison provinces were Manitoba and Saskatchewan (unless stated otherwise). Heteroskedasticity-consistent HC3 SEs are used.

^b^ Manitoba, Saskatchewan, Alberta, and Nova Scotia, Canada.

^c^ Ontario, Manitoba, Saskatchewan, Alberta, Nova Scotia, and New Brunswick, Canada.

## Discussion

To our knowledge, this study provides the first evaluation of the association of British Columbia’s Safer Opioid Supply policy with opioid prescribing and opioid-related health outcomes at the population level. We obtained both graphical and regression evidence that the policy was associated with increases in prescriptions and claimants of opioids targeted by the policy. However, the policy was also associated with an increase in hospitalizations, albeit with no statistically significant increases in deaths.

What could explain the higher hospitalization rate after the policy’s implementation? One potential reason is that participants in British Columbia’s Safer Opioid Supply policy program diverted safer opioid supply for various reasons, including to purchase unregulated fentanyl.^29,30^ It is also possible that a higher supply of prescription opioids led to an increase in prescription opioid misuse, which in turn, could increase hospitalization risks. Another possibility is that availability and/or toxicity of an unregulated drug supply increased more in British Columbia than in comparison provinces, leading to more hospitalizations in British Columbia. While we do not have data on unregulated drug supply, our regression analyses controlled for this potential difference by including province-specific time trends. It may also be argued that more hospitalizations and deaths were due to increased toxicity of unregulated opioids and reduced access to harm-reduction services during the COVID-19 pandemic. However, our analyses showed that the observed increases in hospitalizations and deaths were even greater after excluding the COVID-19 pandemic washout period, and in particular, we found evidence of a positive dose-response relationship after the policy’s expansion.

The increase in prescription rates without a significant increase in prescriber rates suggests that a small number of prescribers contributed to the increased prescriptions. While this might reflect hesitancy among physicians to participate in the Safer Opioid Supply policy program^9,31^ and possible frequent prescriptions of small opioid amounts, it is important to ensure that safer supply opioids are prescribed to and used by people who use drugs and are targeted by the policy. In particular, given some reports of diversion of safer supply opioids, measures to address such diversion (eg, witnessing ingestion or injection of the drug by a health professional^32^) are needed.

It is worth noting that British Columbia has a high incidence of overdose deaths and a long history of harm-reduction approaches (eg, provision of supervised consumption sites). While these differences were controlled for by the province-specific fixed effects and time trends in our DD analyses, they might explain why there was no significant increase in deaths after the policy. It will be interesting to see how death rates will play out in other contexts if safer supply is offered in the absence of supervised consumption sites and easy access to naloxone. Also, as British Columbia decriminalized possession of small amounts of illicit drugs in January 2023, it will be useful to assess the combined effects of these 2 policies in future studies in terms of both uptake of safer supply and resulting health outcomes.

There is a broad consensus that high rates of opioid prescribing during the 1990s have been the driver of the present-day opioid crisis. The Safer Opioid Supply policy is a response to the crisis and aims to reduce opioid overdose by inducing opioid users to switch from illegal to legal opioids. Our finding of higher rates of hospitalization during the first 2 years of implementation of the Safer Opioid Supply Policy is potentially concerning and suggests a need to carefully monitor how safer supply approaches influence opioid use, addiction, and overdose in the long term.

### Limitations

This study has several limitations. First, we used only Manitoba and Saskatchewan as comparison provinces, as data on prescription outcomes were available for only these 2 provinces. However, sensitivity analyses including other provinces and using the synthetic DD approach indicated that our results were robust. Second, as the drugs under consideration could also be used for other clinical conditions, we were unable to attribute the observed increase in prescriptions exclusively to the policy. However, we are aware of no other factors occurring around the time of policy implementation that may have led to increased demand for these drugs for pain management or other conditions. Third, there were prepolicy quarter-by-quarter fluctuations in hospitalizations and deaths, which may have been driven by a variety of changes in supply-side factors (eg, prescribing practices and illicit opioid supply) and demand-side factors (eg, patients’ awareness of opioid harms and illicit opioid prices). Although the prepolicy trends were broadly similar between British Columbia and the comparison provinces, future work that uses longer-term data to discern meaningful trends would be helpful.

Another limitation is that we were unable to examine heterogeneity in the policy effects due to lack of consistent aggregate-level data across demographic groups. It is possible that certain subgroups (eg, polysubstance users or those with higher opioid tolerance levels) continue to be at high risk of using unregulated opioids (and thus have higher risk of hospitalization and death), while the risk of death is reduced for other subgroups that switch to safer supply. Future studies using individual-level data^33^ can examine this heterogeneity. Last, with a small number of provinces, we were unable to account for within-province correlation in outcomes using clustering procedures suitable for a small number of clusters, which could result in a downward bias in SEs and a higher likelihood to find statistically significant results. However, the large magnitude of the observed differences (especially the increases in opioid prescriptions and hospitalizations) and the graphical evidence of changes after policy implementation suggest that the association between the policy and outcomes is likely real.

## Conclusions

Two years after its launch, the Safer Opioid Supply Policy in British Columbia was associated with higher rates of prescribing of opioids but also with a significant increase in opioid-related hospitalizations. These findings may help inform ongoing debates about this policy not only in British Columbia but also in other jurisdictions that are contemplating it.

## Notes

Supplement 1. **eMethods.** Details of Statistical Analyses **eFigure 1.** Unadjusted Trends in Outcomes, British Columbia vs 4 Control Provinces **eFigure 2.** Unadjusted Trends in Outcomes, British Columbia vs 6 Control Provinces **eTable 1.** Subgroup Analyses for Prescription Outcomes
eReferences

Supplement 2.
Data Sharing Statement
