# Supplementary material for: British Columbia’s Safer Opioid Supply Policy and Opioid Outcomes
Source: JAMA Intern Med. 2024 Jan 16;184(3):256–64. doi: 10.1001/jamainternmed.2023.7570 (PMC10792500; doi:10.1001/jamainternmed.2023.7570)
Supplement: Supplement 1. — eMethods. Details of Statistical Analyses eFigure 1. Unadjusted Trends in Outcomes, British Columbia vs 4 Control Provinces eFigure 2. Unadjusted Trends in Outcomes, British Columbia vs 6 Control Provinces eTable 1. Subgroup Analyses for Prescription Outcomes eReferences [file jamainternmed-e237570-s001.pdf]

Supplementary Online Content

Nguyen HV, Mital S, Bugden S, McGinty E. British Columbia's Safer Opioid Supply policy and opioid outcomes. *JAMA Intern Med*. 2024;184(1.3):e237570. doi:1001/jamainternmed.2023.7570

eMethods. Details of Statistical Analyses

eFigure 1. Unadjusted Trends in Outcomes, British Columbia vs 4 Control Provinces

eFigure 2. Unadjusted Trends in Outcomes, British Columbia vs 6 Control Provinces

eTable. Subgroup Analyses for Prescription Outcomes

eReferences

This supplementary material has been provided by the authors to give readers additional information about their work.

eMethods: Details of statistical analyses

The difference-in-differences (DD) analyses were implemented using the following regression model:

$$Y_{pt} = \alpha + \beta_1(Safer\ Supply\ Policy)_{pt} + \beta_2 Z_{pt} + \beta_3 Province_p + \beta_4 Time_t + \beta_5\ Province_p \times Time\ Trend_t + errors$$

where Y_{pt} was the outcome of interest in province p in time t (quarter-year). The covariate of interest was *Safer Supply Policy*$_{pt}$, an indicator equal to 1 if the policy was in effect in a province (i.e., when p=British Columbia and t=after quarter 1 of 2020), and 0 otherwise. The coefficient of interest was β_1. Z_{pt} was a vector of time-varying provincial-level covariates, including proportion of children aged 0-17 years, proportion of males, consumer price index, and unemployment rate. As the policy was implemented during the COVID-19 pandemic and public health COVID restrictions could affect availability and use of opioids as well as provision of addiction and mental health services[1,2], our model also controlled for public health COVID-19 restrictions (using the COVID-19 stringency index developed by Bank of Canada[3]) that vary both over time and across provinces. The regressions also included province indicators ($Province_p$) to control for all time-invariant characteristics of provinces and quarter-year indicators ($Time_t$) to control for secular changes or shocks in outcomes that are common to British Columbia and the comparison provinces. Additionally, we included province-specific linear time trends ($Province_p \times Time\ Trend_t$) to control for possible differences in trends across provinces.

We estimated the regressions by ordinary least square (OLS) and calculated heteroskedasticity-consistent HC3 standard errors. All analyses were conducted at province-quarter level using Stata 18 software. Tests were two-sided, and a 5% significance level was used.

We conducted several analyses to investigate the robustness of our results. First, as the policy's launch coincided with the onset of the COVID-19 pandemic, we conducted additional analyses to rule out confounding effects of the pandemic. We first re-ran the analysis excluding the 'COVID-19 washout period' between quarter 2 of 2020 and quarter 1 of 2021. In this period, pandemic-related public health restrictions were at their peak and significantly reduced access to harm reduction facilities, which could increase hospitalizations and deaths. If any observed changes in hospitalizations and deaths were due to the pandemic, we would expect to see no or smaller changes in these outcomes after dropping the peak pandemic period. Second, we examined the policy effects separately during the first year (i.e., the policy's launch) and the second year (i.e., the policy's expansion). A larger policy effect during the policy expansion would indicate a dose-response relationship and suggest that observed outcome changes would be more likely attributed to the policy than the pandemic.

Second, we examined the sensitivity of our results to exclusion of province-specific linear time trend, demographic controls, and the COVID-19 stringency index. Third, to examine whether the results were sensitive to choice of comparison provinces, we additionally included Alberta and Nova Scotia as comparison provinces. Next, we conducted an event study analysis in which we replaced the indicator for policy exposure with a series of event time indicators relative to the timing of the policy adoption in British Columbia. Finally, to examine the robustness of our results to potential violation of the parallel trends assumption, we used the synthetic DD method that reweights control groups and time periods to ensure that outcome trends are parallel between control and treatment groups. As this method requires a sufficient pool of provinces to form a synthetic control group, we included not only Manitoba, Saskatchewan, Alberta, and Nova Scotia but also Ontario and New Brunswick as comparison provinces. (There were safer opioid supply programs in Ontario and New Brunswick but these were small scale pilot studies and implemented in only 9 (out of 101) community health centers in Ontario and only one recovery centre in New Brunswick

between 2020 and 2021[4]). In these analyses with additional comparison provinces, we looked at only hospitalization and death outcomes as data on prescription outcomes were not available for these provinces. (The remaining provinces were not used as comparison provinces because Newfoundland and Labrador and Prince Edward Island had too few observations, and data for Quebec were not available.)

eFigure 1: Unadjusted trends in outcomes, British Columbia vs 4 control provinces

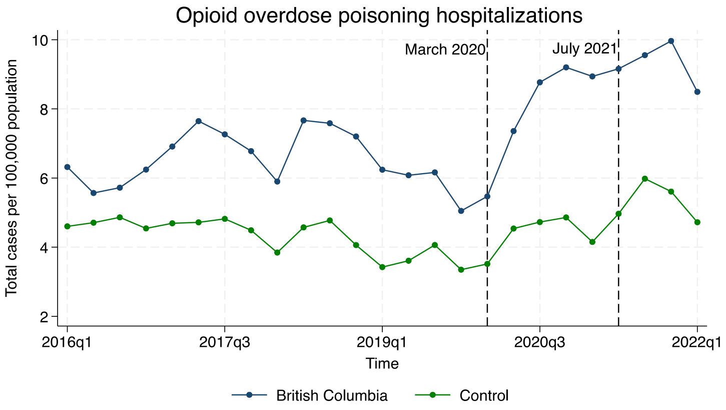

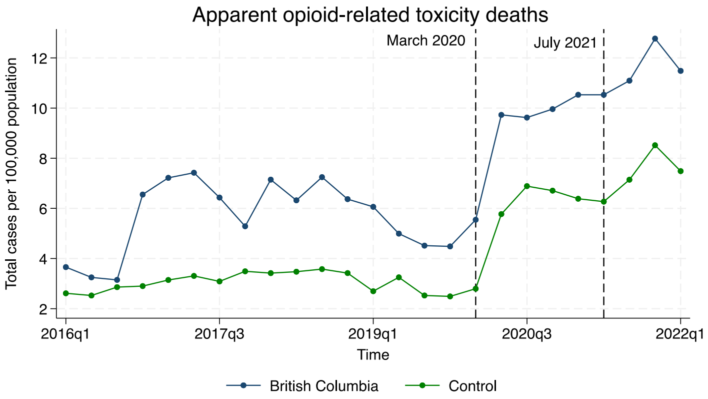

Note: Control group includes 4 provinces (Nova Scotia, Manitoba, Saskatchewan, and Alberta). The Safer Opioid Supply policy was implemented in March 2020 and expanded in July 2021.

eFigure 2: Unadjusted trends in outcomes, British Columbia vs 6 control provinces

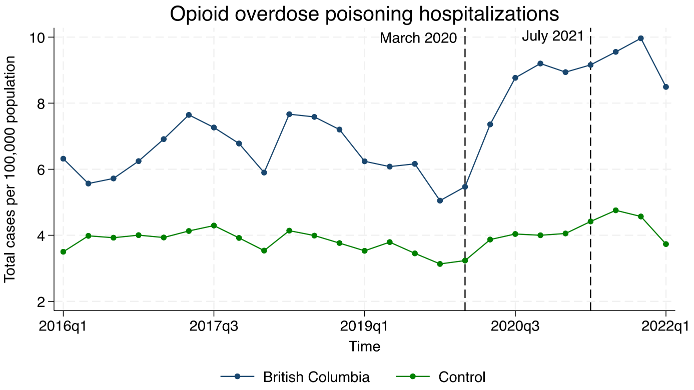

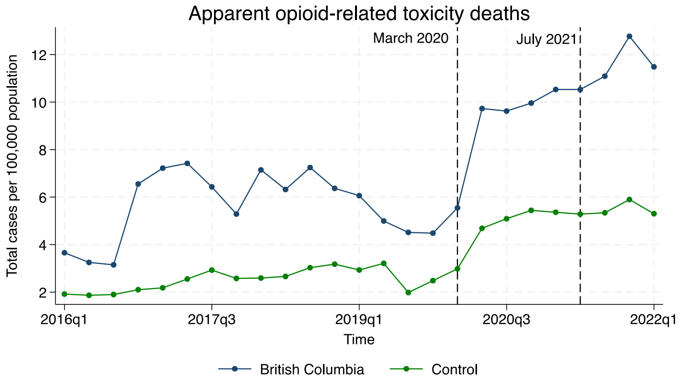

Note: Control group includes 6 provinces (Nova Scotia, Manitoba, Saskatchewan, Alberta, Ontario and New Brunswick). The Safer Opioid Supply policy was implemented in March 2020 and expanded in July 2021.

eTable Subgroup analyses for prescription outcomes

Outcomes (No./100,000 population)	Difference-in-Differences estimates	95% CI	*p*-value
Females			
Prescription rate (N=75)	714.7	(280.7 – 1148.8)	0.002
Claimant rate (N=75)	67.2	(-17.7 – 152.2)	0.12
Prescriber rate (N=75)	13.0	(-2.9 – 29.0)	0.11
Males			
Prescription rate (N=75)	1874.5	(991.0 – 2758.1)	<0.001
Claimant rate (N=75)	95..0	(27.6 – 162.4)	0.007
Prescriber rate (N=75)	10.0	(-1.5 – 21.6)	0.09
Age <25			
Prescription rate (N=49)	55.5	(-98.5 – 209.6)	0.45
Claimant rate (N=49)	-4.7	(-64.2 – 54.8)	0.87
Prescriber rate (N=49)	0.1	(-14.7 – 14.9)	0.99
Age 25-64			
Prescription rate (N=51)	1820.6	(-413.3 – 4054.5)	0.103
Claimant rate (N=51)	106.0	(-53.5 – 265.5)	0.18
Prescriber rate (N=51)	-1.8	(-32.9 – 29.3)	0.9
Age 65+			
Prescription rate (N=75)	87.2	(-93.9 – 268.3)	0.34
Claimant rate (N=75)	33.5	(-9.6 – 76.6)	0.12
Prescriber rate (N=75)	10.3	(0.2 – 20.5)	0.046

Data are from quarter 1 of 2016 to quarter 1 of 2022. Rates are numbers per 100,000 population. Estimates are from difference-in-difference regressions estimated using ordinary least squares and control for proportion of children aged 0-17 years in the population, proportion of males, consumer price index, unemployment rate and covid restriction score in the province, province and quarter-year fixed effects, and province-specific linear time trend. Control provinces are Manitoba and Saskatchewan. Heteroskedasticity-consistent HC3 standard errors are used.

References

1. Canadian Institute for Health Information. Unintended Consequences of COVID-19 Impact on Harms Caused by Substance Use. Published 2021. Accessed July 13, 2023. https://secure.cihi.ca/free_products/unintended-consequences-covid-19-substance-use-report-en.pdf

2. Ontario COVID-19 Science Advisory Table. The Impact of the COVID-19 Pandemic on Opioid-Related Harm in Ontario. doi:10.47326/ocsat.2021.02.42.1.0

3. Bank of Canada. COVID-19 stringency index. Published 2023. Accessed June 17, 2023. https://www.bankofcanada.ca/markets/market-operations-liquidity-provision/covid-19-actions-support-economy-financial-system/covid-19-stringency-index/

4. Government of Canada. Interactive map: Canada's response to the opioid crisis. Published May 22, 2018. Accessed February 23, 2022. https://health.canada.ca/en/health-canada/services/drugs-medication/opioids/responding-canada-opioid-crisis/map.html
