# Supplementary material for: British Columbia’s Safer Opioid Supply Policy and Opioid Outcomes
Source: JAMA Intern Med. 2024 Jan 16;184(3):256–64. doi: 10.1001/jamainternmed.2023.7570 (PMC10792500; doi:10.1001/jamainternmed.2023.7570)
Supplement: Supplement 2. — Data Sharing Statement [file jamainternmed-e237570-s002.pdf]

Data Sharing Statement

Nguyen. British Columbia’s Safer Opioid Supply Policy and Opioid Outcomes. *JAMA Intern Med*. Published January 16, 2024. doi:10.1001/jamainternmed.2023.7570

Data
Data available: No
